# Degradation pathways and chemical stability of regenerated cellulose fiber-reinforced bio-polyamide 5.10 composites under acidic and alkaline conditions

**DOI:** 10.1038/s41598-025-23295-2

**Published:** 2025-10-09

**Authors:** Celia Katharina Falkenreck, Jan-Christoph Zarges, Hans-Peter Heim

**Affiliations:** Institute of Materials Engineering, Plastics Engineering, University of Kassel, Moenchebergstr. 3, 34125 Kassel, Germany

**Keywords:** Bio-polyamide, Regenerated cellulose fibers, Fiber-matrix bond, Durability, Chemistry, Engineering, Materials science

## Abstract

Bio-based polyamides (PA) represent an emerging class of engineering biopolymers that combine high performance with renewable origins. In particular, PA5.10, derived from renewable monomers, offers an attractive alternative to petroleum-based matrices in sustainable composite applications. To advance the understanding of its environmental stability, this study explores the chemical resistance and degradation pathways of neat PA5.10 and regenerated cellulose fiber (RCF)-reinforced PA5.10 (20 wt%). Standardized specimens were prepared by twin-screw extrusion and injection molding and subsequently exposed to aqueous, acidic, alkaline, and organic environments for up to 168 h. Characterization included tensile testing, Fourier-transform infrared spectroscopy (FTIR), melt volume rate (MVR), moisture uptake, and scanning electron microscopy (SEM). Hydrolytic and chemical aging caused chain splitting processes, fiber swelling, and fiber-matrix debonding, as evidenced by viscosity reduction, altered FTIR spectra, and mechanical deterioration. The composites exhibited pronounced sensitivity in acidic and alkaline media, with moisture uptake further reducing tensile strength and modulus. These findings provide new insights into the chemical stability of bio-based polyamides and highlight key challenges for their long-term use in sustainable composite applications. Addressing these limitations through targeted material design will be essential to expand the application range of RCF-reinforced bio-based polyamides in automotive and engineering sectors.

## Introduction

In recent years, the development of sustainable materials has advanced significantly, leading to the integration of natural fibers into polymers to enhance mechanical properties while reducing the CO_2_-footprint^1,2^. Natural fiber-reinforced bio-based polyamides have emerged as promising candidates in this context, offering a balance between high mechanical properties, especially in contrast to other bio-based polymers like polylactide, and sustainability^3–5^. Natural fiber-reinforced composites (NFC) therefore present a lightweight material solution for components such as interior panels, engine covers, and structural reinforcements, providing benefits such as reduced weight and lower CO₂ emissions compared to glass fiber-reinforced composites (GFC). However, the durability of NFC remains a critical area of investigation, particularly in the automotive industry, where the polymer components are exposed to diverse chemicals as well as several environmental stresses in form of high temperatures or humidity. Bio-based polyamides, like PA5.10 or PA10.10, derived from renewable resources like castor oil or corn starch, are gaining attention as an alternative to conventional petroleum-based polyamides, like PA6. While they offer comparable mechanical properties, differences in chemical structure and crystallinity influence their moisture absorption and aging behavior, in case of PA5.10, due to longer molecular chains^6,7^. Compared to a petroleum-based PA6, PA5.10 exhibit slower rates of moisture absorption and a higher chemical resistance, positively impacting its long-term stability.

**Fig. 1:**
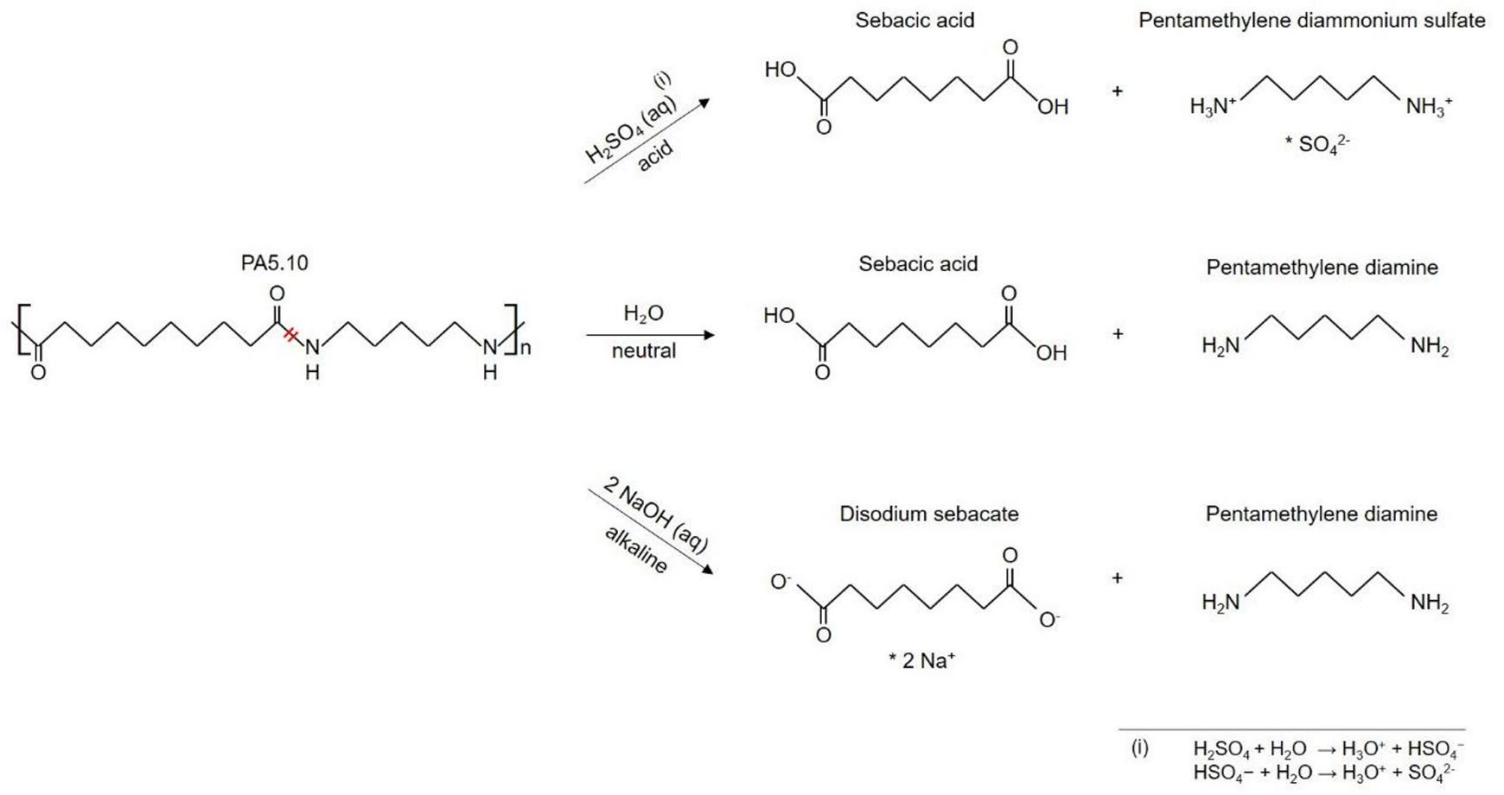
Molecular chain splitting processes of PA5.10 due to acid, neutral and alkaline environment. In acidic environment, SO_4_ is split off to form sebacic acid and pentamethylene diammonium sulphate. In water, PA5.10 splits into sebacic acid and pentamethylene diamine, reversing the polycondensation. In alkaline environments, disodium sebacate and pentamethylene diamine are formed by splitting off two natrium molecules.

Aliphatic polyamides, produced by polycondensation, are synthesized by the reaction of a carboxylic acid with an amine by the elimination of water^8^. As it is an equilibrium reaction, hydrolysis is a key concern, particularly under aqueous conditions, as it leads to molecular chain splitting, reducing the polyamide´s molecular weight, as it can be seen in Fig. 1^9^. As moisture migrates into the amorphous areas of the hygroscopic polyamide, the increased free volume enables fluid diffusion and promotes molecular chain splitting processes. This results in reduced mechanical properties, including a decreasing tensile strength, stiffness, and elongation at break. Acidic conditions, such as an aqueous sulfuric acid solution (e.g. simulated acid rain), can further accelerate degradation by catalyzing the breakdown of amide bonds due to the formation of H_3_O^+^^10–13^. The molecular chain of PA5.10 splits in a similar way to hydrolytic chain splitting in aqueous environment, but other cleavage products are formed which is visualized in Fig. 1. Furthermore, an alkaline environment, like sodium hydroxide solutions, intensifies degradation through base-catalyzed reactions, attacking both the polymer matrix and fiber-matrix interface, often leading to embrittlement as well as to a loss in single fiber tensile strength and fiber-matrix-adhesion. Furthermore, the alkaline degradation of polyamide results in degradation products like sodium salts which can lead to a formation of salt crystals on the polyamides surface. These crystals can change the melt viscosity as they do not melt properly under high temperatures, negatively affecting the polymer´s melt flow^10,14^.

**Fig. 2:**
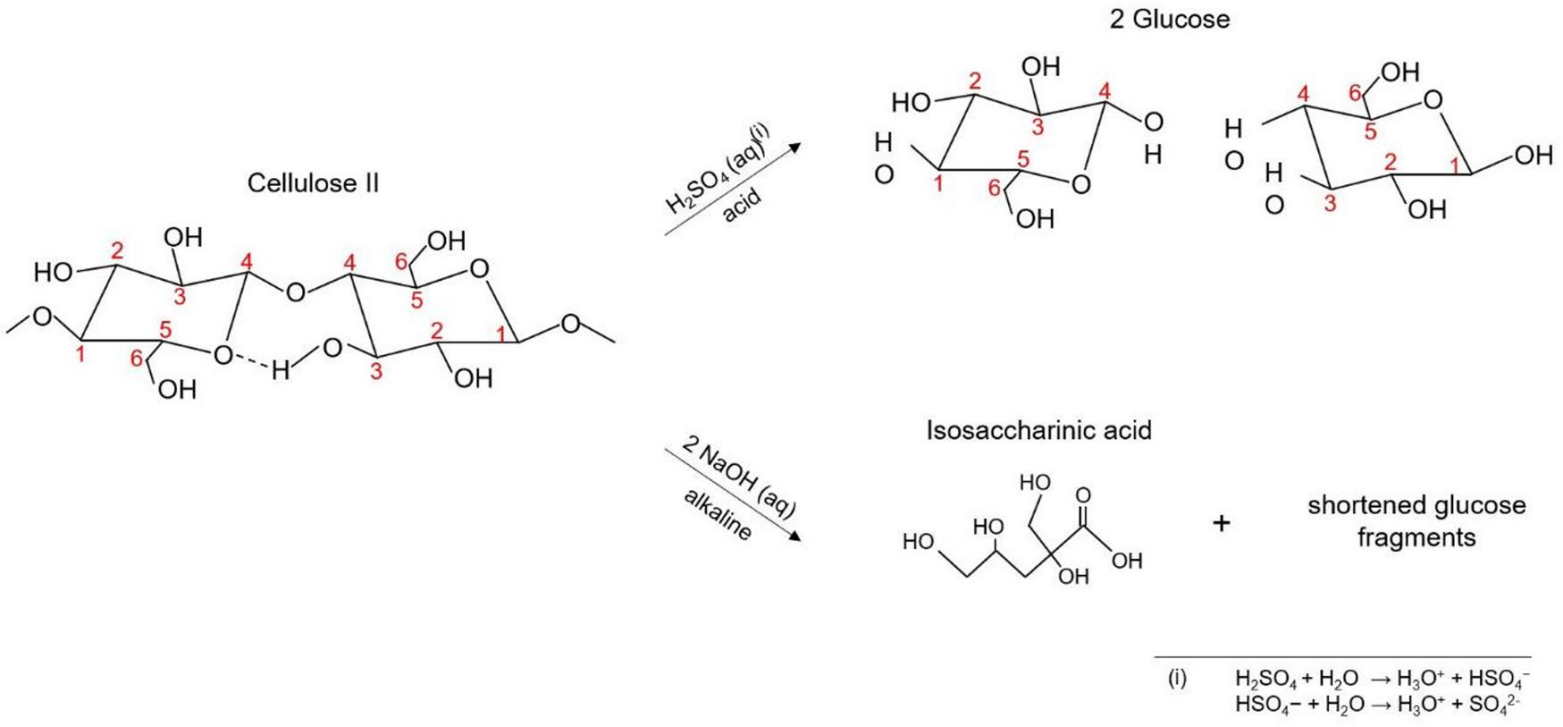
Molecular chain splitting of the β-1,4-glycosidic bonds due to acid environment and peeling-off effect induced by alkaline solution. In acidic environment, the cellulose II chain is split into individual glucose units. In alkaline environment, the molecular chain of cellulose II breaks down into isosaccharinic acid and several shorter glucose fragments.

In contrast to glass fibers (GF), chemically produced regenerated cellulose fibers (RCF), used for automotive applications, e.g. to reinforce tires, show a hygroscopic behavior, making them susceptible to moisture absorption and swelling. However, the effect is not as severe as with natural jute or hemp fibers, as RCF are chemically synthesized and do not contain any share of lignin^15^. Therefore, they have reproducible and adjustable properties and are not influenced by growth batch fluctuations. That aside, temperature sensitivity as well as formation of agglomerates in injection molded components are the main downsides of composites with cellulose-based fibers^16^. In automotive applications, where structural reliability is essential, RCF therefore have a downside compared to GF^17^. Regenerated cellulose, the so called cellulose II, has two different chain ends due to its specific molecular structure. The reducing end contains a free aldehyde group, while the non-reducing end is capped with a hydroxymethyl group. These two ends behave differently during chemical reactions, with the reducing end being more affected by degradation, while the non-reducing end is more stable in alkaline or acid environment^18^. This structural difference has an important role in cellulose’s reactivity and on the two distinct degradation mechanisms. There is the peeling-off reaction on one hand, where glucose units are sequentially removed from the reducing end, and internal nucleophilic substitution on the other hand, which occurs when hydroxide ions initiate a reaction at the β-1,4-glycosidic bonds within the cellulose chain, leading to molecular chain splitting^18,19^. Therefore, an exposure to acids or alkaline solutions weaken the RCF by disrupting hydrogen bonds as well as increasing molecular chain splitting processes as demonstrated in Fig. 2. This reduces structural integrity of the cellulose II, resulting in a reduced single fiber tensile strength and embrittlement. For this reason, instead of a fiber pull-out, the fibers rupture under tensile stress, which can be seen on the fracture surfaces of the composite e.g. in SEM images. Organic solvents such as ethanol and 2-propanol act primarily as plasticizers. These substances, especially the ethanol, lower the glass transition temperature on one hand and increase chain mobility in the amorphous phase on the other hand. As there is a formation of hydrogen bonds between the hydroxyl group of the ethanol and the amide, the ethanol absorbs into the polymer´s structure. Additionally, after ethanol evaporation, e.g. due to standard climate conditioning, hydrogen bonding sites are accessible for water absorption, further enhancing moisture absorption^20,21^. Hydrophobic fluids, such as engine oil, interact less with the polyamide or fibers due to their low polarity. Nonetheless, long-term exposure can cause softening or swelling of the polyamide^11,22^.

The literature already contains a broad range of information on the chemical resistance of PA6 and cellulose observed separately. For this reason, this study aims to analyze how this knowledge can be transferred to the new bio-based PA5.10 and how it interacts with the cellulose-based reinforcing fibers. Therefore, this study systematically evaluates the effects of various chemicals and fluids on RCF-reinforced PA5.10 and aims to provide insights into the suitability and chemical resistance. The findings will contribute to the development of durable, high-performance, and eco-friendly composite solutions for the automotive sector.

## Materials and methods

### Materials

A 100% bio-based polyamide 5.10 (AKROMID^®^ NEXT 5.10 3 EXP nature) was provided by the company AKRO-COMPOUNDS GmbH (Niederzissen, Germany). Its raw material is synthesized from corn starch and castor oil. The bio-based and biodegradable regenerated cellulose fiber (RCF) Rayon from Cordenka GmbH & Co. KG (Obernburg am Main, Germany) is made using the viscose process. The chopped short fibers have a length of approximately 2.5 mm and an average fiber diameter of 12.65 μm. The properties of PA5.10 and the RCF can be seen in Table 1.

**Table 1:** Properties of PA5.10 (values from self-conducted research and^7,23,24^.

	Density in g/cm^3^	Melt temperature in °C	Maximum saturation point in %	Tensile strength in MPa	Young’s modulus in GPa
PA5.10	1.08	217	4.5	43.5	2.2
RCF	1.5	–	7.6	830	20

### Compounding

Prior to compounding, PA5.10 was pre-dried to a moisture content below 0.1 wt% using a Dry Jet Easy desiccant dryer (TORO-Systems, Gfk Thomas Jakob und Robert Krämer GbR, Igensdorf, Germany) at 80 °C for 4 h. The RCF were conditioned separately in an air convection oven at 105 °C for 24 h to ensure low residual moisture, as they cannot be processed when moist. The composite formulation of PA5.10RCF20 consisted of 20 wt% RCF. This corresponds to approximately 13 vol%, aligning with the volumetric fiber content found in typical GF-reinforced composites with 30 wt% GF, commonly used in the automotive sector. Compounding was carried out using a ZSE 18 HPe co-rotating twin-screw extruder (Leistritz Extrusionstechnik GmbH, Nuremberg, Germany), featuring 18 mm screw diameter and a length-to-diameter ratio of 40D. The feeding zone up to the 20D position, located upstream of the side feeder, was configured with conveying elements designed for enhanced volume capacity, along with kneading zones for improved material homogenization. Beyond the side feeding point, where the RCF was incorporated, only standard conveying elements were used to minimize fiber damage. Both the main and side feeder screws were operated at a rotational speed of 200 rpm. The matrix and RCF feed rates were regulated via gravimetric dosing units (Brabender Technology GmbH & Co. KG, Duisburg, Germany). To mitigate the risk of thermal degradation of the cellulose fibers, compounding temperatures were kept as low as possible, according to the material-specific limits outlined in Table 2^25,26^. Post-extrusion, the composite strands were cooled using a compressed air conveyor system and pelletized with a strand cutter (Scheer SGS 25.E, Maag Germany GmbH, Grossostheim, Germany), resulting in pellets with lengths ranging from 3 to 4 mm.

**Table 2:** Temperature setpoints of the compounding process of PA5.10RCF20.

Zone	Feeding zone	1	2	3	4	5	6	7	Nozzle
Temperature in °C	23	230	230	220	220	220	210	210	230

### Injection molding

Type 1 A test specimens (DIN EN ISO 527-2) were produced using the Allrounder 320 C injection molding machine (Arburg GmbH & Co. KG, Lossburg, Germany) using a screw diameter of 25 mm. Prior to injection molding, the PA5.10 as well as the PA5.10RCF20 granules were dried at 80 °C for 4 h, to avoid residual moisture. The temperature profile of the injection molding process can be seen in Table 3. The type 1 A injection mold included a cold runner and two cavities. The total cycle time was 70 s, comprising a 21 s packing phase at 550 bar and a 35 s cooling phase. The injection pressure was in between 350 and 400 bar (PA5.10) and 1400–1500 bar (PA5.10RCF20).

**Table 3:** Temperature setpoints of the injection molding process of PA5.10.

Zone	Feeding zone	1	2	3	4	5	Nozzle	Mold
Temperature in °C	23	230	230	235	235	240	240	40

### Storage of the type 1 A test specimens

Injection molded type 1 A test specimens (PA5.10 and PA5.10RCF20) were stored in various chemicals and fluids for 168 h in closed glass containers under a fume hood. The selection of the chemicals and fluids were made on the basis of ISO 16750-5 and conventional automotive chemicals. A detailed list of these can be found in Table 4.

**Table 4:** Chemicals and fluids used for storage of the 1 A test specimens.

Condition	Comment
As-molded	Reference batch
Distilled water	
Salt water	2.5% NaCl
Soap water	0.1%
Rubbing alcohol	
Engine oil	
Acid rain	Sulfuric acid 0.0005 mol/ l, pH 4.5
Sodium hydroxide solution	40% NaOH
Ethanol	> 99%
2-Propanol	> 99%

Subsequent to the chemicals/fluid storage, the specimens were conditioned at standard climate (23 °C, 50%rH) for another 168 h, according to ISO 291^27^. As-molded neat and RCF-reinforced PA5.10 type 1 A test specimens were used as reference specimens.

### Determination of the moisture absorption

The absorbed moisture c (1) of 15 type 1 A test specimens per condition were determined using the electronic precision scale (CUBIS II, Sartorius AG, Goettingen, Germany) with 10^− 3^ g accuracy.

1 $$\:c=\:\frac{{w}_{t2}-{w}_{t1}}{{w}_{t1}} \times 100\:\%$$

w_t1_ is the weight of the as-molded specimens and w_t2_ is the specimens weight after the chemical/fluid storage and 168 h of conditioning at standard climate.

### Scanning electron microscopy (SEM)

The surface morphology of selected RCF-reinforced specimens was investigated with SEM images (MV2300, CamScan Electron Optics Services, Ottawa, Canada). Imaging was performed at an acceleration voltage of 10 kV and a magnification of 1000×. To enable proper visualization, the fracture surfaces of the type 1 A specimens were gold-coated via sputtering prior to analysis.

### Fourier transform infrared spectroscopy (FTIR)

FTIR spectroscopy in attenuated total reflection (ATR) mode was used to examine the chemical structure of the neat PA5.10 type 1 A test specimens. Analyses were carried out under standard laboratory conditions (23 °C, 50%rH) in the spectral range of 4000–800 cm^−1^. For each condition, three specimens were measured using an IRAffinity-1 S spectrometer (Shimadzu, Kyōto, Japan) equipped with a germanium crystal.

### Differential scanning calorimetry (DSC)

The DSC Q 1000 module from TA Tools (New Castle, USA) was used to measure the crystallinity of the non-reinforced specimens in this study. Two specimens of each condition were heated at 20 K/min until the melting temperature was reached. The first melting cycle was used to analyze the effects of aging, as changes in crystallinity and microstructure are visible in the first melting peaks. The integral below the peak of the cycles corresponds to the melt-enthalpy. It can be used to calculate the crystallinity by dividing it by the value of the 100% crystallinity of polyamide, with a known literature value of 208 J/g^7^.

### Melt volume rate (MVR)

The melt volume rate was determined using the Meltflixer 2000 by Thermo Haake (Karlsruhe, Germany), following ISO 1133-1. Tests were performed with two specimens per condition at 250 °C with a 2.16 kg load. Measurements were carried out on neat PA5.10, as the presence of fibers can interfere with the viscosity and comparability of the melt flow results.

### Tensile test

Quasi-static tensile tests (*n* = 5) were conducted in accordance with DIN EN ISO 527 on type 1 A injection-molded specimens under standard climatic conditions (23 °C, 50%rH) using a Shimadzu AGS-X universal testing machine (Kyōto, Japan), equipped with a 5000 N load cell with a gauge length of 122 mm. A contact extensometer was attached directly to the specimen to measure strain during loading. A test speed of 5 mm/min was applied for RCF-reinforced composites, while a higher speed of 25 mm/min was used for neat PA5.10 to improve testing efficiency, as it reaches its maximum tensile strength at a slower rate. All specimens were elongated up to a maximum strain of 80%.

### Statistical analysis

All measurement results were tested against each other for statistical significance using ANOVA in Origin2021b (OriginLab Corporation, Northampton, USA), with a level of significance of 0.05. This ensured that any observed differences between the data sets were meaningful rather than due to random variation. Only those differences that passed the significance threshold were considered for further analysis.

## Results and discussion

### Moisture absorption

**Fig. 3:**
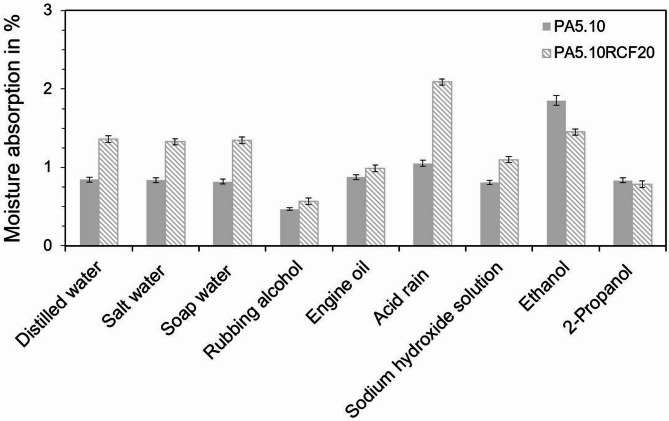
Moisture absorption of the type 1 A test specimens subsequent to 168 h of chemical/fluid storage and conditioning. It can be seen that the RCF-reinforced type 1 A test specimens absorb significantly more moisture in aqueous solutions than the neat PA5.10, due to the hygroscopic material behavior of the RCF. Storage in acid rain further accelerates the moisture absorption in the RCF composites. In addition, a very high moisture absorption of PA5.10 in ethanol can be observed.

Starting with a consideration of the moisture absorption of PA5.10 and PA5.10RCF20 subsequent to the storage in the chemicals/fluids, Fig. 3 shows the moisture absorption in percent in relation to the weight of the as-molded type 1 A test specimens. For all specimens, apart from the ones stored in the organic solvents ethanol and 2-propanol, the moisture absorption of the neat PA5.10 is lower than of the PA5.10RCF20 specimens. The increased moisture content of the RCF-reinforced specimens results from the elevated moisture absorption of RCF compared to the hygroscopic polyamide^7^. In contrast, RCF do not absorb moisture in ethanol and 2-propanol, as the organic solvents prevent the water molecules from binding, due to their high polarity^28^.

Nonetheless, the PA5.10 absorbs a significant amount of water subsequent to the storage in ethanol. This strong moisture uptake of ethanol in polyamides results from hydrogen bonding between the hydroxyl group of ethanol and the polyamide´s amide groups, facilitating ethanol absorption into the polymer structure. After ethanol evaporation, due to the subsequent conditioning at standard climate for 168 h, the hydrogen bonding sites are accessible for water molecules, further enhancing moisture absorption. However, the moisture absorption of the 2-propanol stored specimens is not that pronounced due to its lower polarity and steric effects^20,21^. Since the polyamide share in the PA5.10RCF20 is lowered to 80 wt% due to the RCF content, it leads to a correspondingly higher moisture absorption of the neat PA5.10 in both cases.

1A test specimens stored in rubbing alcohol exhibit the lowest moisture absorption, as this is a volatile medium that evaporates after storage, which does not form hydrogen bonds to the polyamide. The specimens stored in engine oil also display a reduced increase in moisture as the oil cannot absorb into the polyamide as fast as water due to its comparatively higher viscosity. The high increase in moisture content of PA5.10RCF20 during storage in acid rain is due to the fact that the acidic environment accelerates the diffusion of water molecules in the amorphous boundary layer of the neat PA5.10 as well as in the RCF^10,11^.

### Morphology and chemical composition

**Fig. 4:**
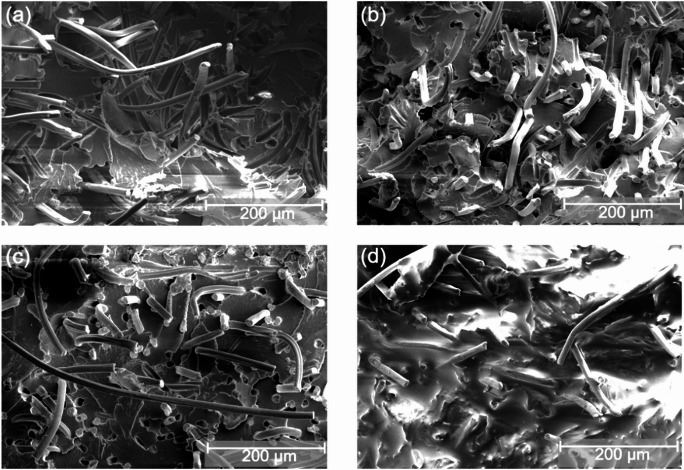
SEM-images of selected type 1A test specimens ((**a**) as-molded, (**b**) ethanol, (**c**) acid rain, (**d**) engine oil) subsequent to 168 h of chemical/fluid storage. Subsequent to storage in ethanol, there is no visual change compared to the as-molded reference. The acid rain causes significantly more fiber ruptures than pull-outs due to a degradation of the fiber. Storage in engine oil results in an oil film covering the entire fracture surface.

SEM images of PA5.10RCF20 in Fig. 4 reveal distinct morphological changes after exposure to ethanol, acid rain and engine oil. In as-molded state (a), the SEM images exhibit numerous well-defined long fiber pull-outs, with only a few fiber fractures. This indicates that the fibers were predominantly pulled-out from the PA5.10 matrix as intact entities, suggesting a low fiber-matrix-adhesion^29–31^. Furthermore, the surrounding matrix appears more ductile, displaying characteristic crack parabolas, which is a typical sign of energy dissipation during deformation^32^. Subsequent to the storage in ethanol (b), no significant decrease in fiber pull-outs or fiber ruptures can be seen, although there was a high moisture absorption visible in Fig. 3. Post-exposure to acid rain (c), the SEM images show more fiber ruptures on the specimens surface. The matrix surrounding the fibers also appears brittle in comparison to the as-molded reference, as the surface is more even. These observations strongly suggest that both the RCF and the PA5.10 matrix suffered hydrolytic degradation in acid environment. This is due to fiber damage resulting because of the chemical exposure on one hand and matrix degradation due to hydrolysis on the other hand^6^. The SEM image (d) of the specimen stored in engine oil shows a persistent oil film on the sample surface. However, the presence of the oil film may obscure certain microscopic details. This finding implies that residual oil remains trapped within the sample, contributing to the measured weight increase after the storage. Moreover, this residual oil is expected to influence the mechanical properties of the material, as further discussed in subchapter 3.3.

**Fig. 5:**
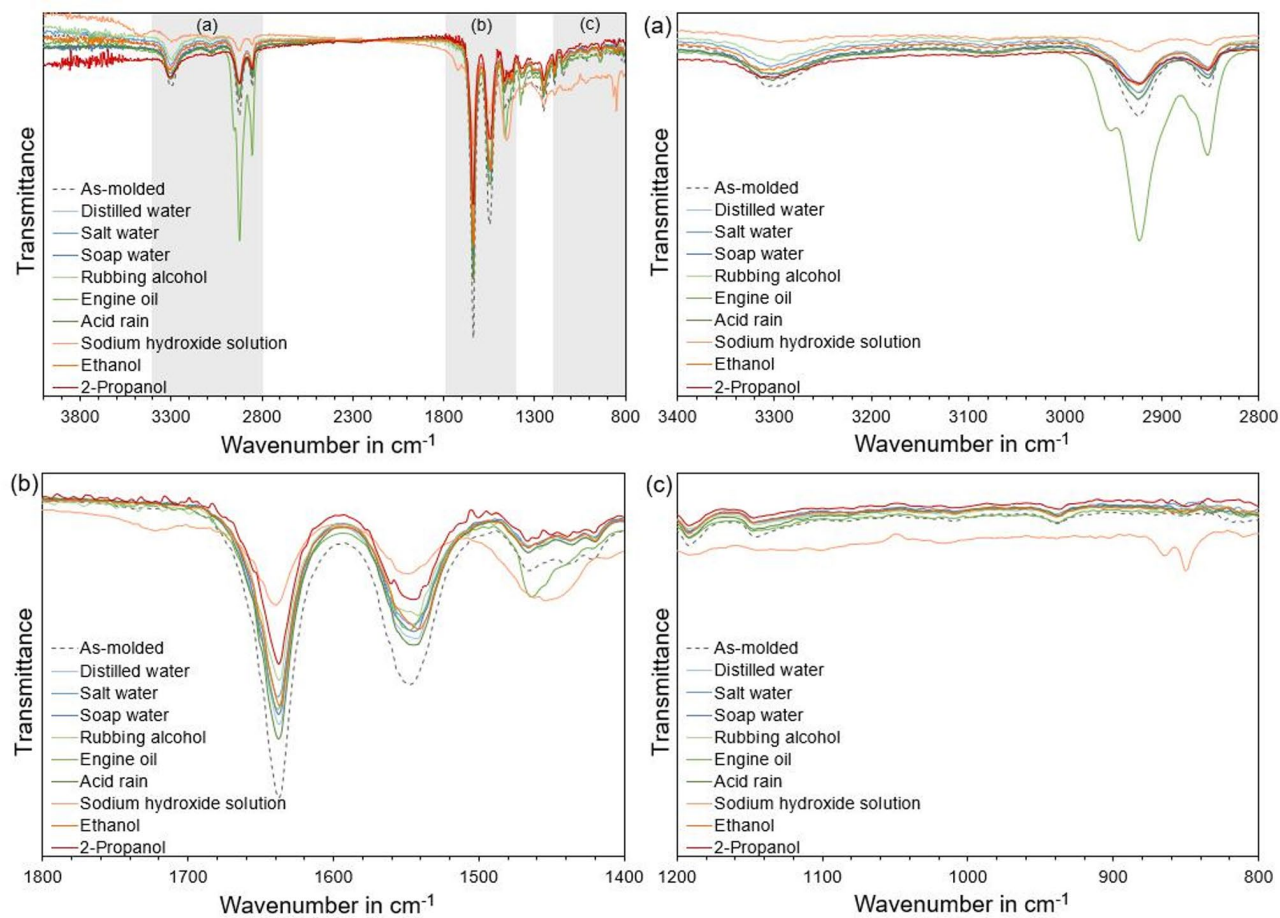
FTIR spectra of neat type 1 A test specimens subsequent to 168 h of chemical/fluid storage and conditioning. The general overview from 4000–800 cm^−1^ can be seen on the top left. The detailed views show the wavenumbers (**a**) 1200–800 cm^−1^, (**b**) 1400–1800 cm^−1^, and (**c**) 3400–2800 cm^−1^. The greatest changes in the spectra can be seen in (**b**) and (**c**) after storage in the sodium hydroxide solution, which results in significant changes in the chemical structure. In (**a**), a reduced transmittance of the specimens can be seen, due to the absorption of the engine oil.

The FTIR measurements were conducted to assess the changes of the chemical composition of neat PA5.10 after exposure to the chemicals/fluids. The transmittance spectrum at the top left of Fig. 5 displays the whole spectrum from 4000 to 800 cm^−1^. For a better visualization, three detailed views are shown in Fig. 5a–c. Figure 5a shows the transmittance spectrum from 3400 to 2800 cm^−1^. A significant difference can be observed subsequent to the storage in engine oil, where the peaks at 2929–2855 cm^−1^ show substantially stronger transmittance peaks compared to the as-molded spectrum. This shift suggests that engine oil induces changes in the polyamide’s molecular structure, likely due to the oil incorporated in the amorphous boundary layer and through the interaction with the polymer’s amide groups, which can lead to the disruption of hydrogen bonding^33^. Furthermore, a decrease in the peaks at 2929–2855 cm^−1^ detect an increase in crystallinity of the polyamide. As the exposure to the sodium hydroxide solution results in high transmittance at 2929–2855 cm^−1^, which can be attributed to the strong alkaline environment, it is an indicator of strong aging processes of the PA5.10, breaking down the amide bonds and consequently increasing the crystallinity, which can also be seen in the results of the DSC in Table 5^33^.

**Table 5:** Degrees of crystallinity of the non-reinforced PA5.10 samples after measurement in the DSC. The highest increase in crystallinity can be seen after storage in soap water, acid rain, 2-propanol, and sodium hydroxide solution.

Condition	Crystallinity in %	
	Mean value	Standard deviation
As-molded	27.300	0.327
Distilled water	29.038	0.436
Salt water	30.649	0.368
Soap water	31.457	0.378
Rubbing alcohol	28.413	0.313
Engine oil	30.543	0.367
Acid rain	31.034	0.372
Sodium hydroxide solution	31.846	0.541
Ethanol	30.702	0.368
2-Propanol	33.173	0.398

In Addition, there might be an interference with sodium salts on the specimens surface which gets detected by the FTIR. With exception of the engine oil stored specimens, all other conditions show an increase in crystallinity compared to the as-molded reference, presumably due to the aging-related degradation of the amorphous areas, as well. Moreover, a decreasing peak at 3300 cm^−1^ indicates an increase in NH_2_ end groups which is a verification for hydrolytic processes^34^. A strong decrease in transmittance can be seen here again with the sodium hydroxide solution, which exhibits the greatest changes in chemical structure.

In Addition, Fig. 5b shows the transmittance spectrum from 1800 to 1400 cm^−1^, which includes the more dominant peaks at 1633 cm^−1^ and 1733 cm^−1^. Due to hydrolytic processes, the molecular chain of the PA5.10 splits in between the C=O and NH bonds, which results in a decrease in NH and methylene groups. A smaller transmittance peak at 1533 cm^−1^ indicates a decrease in NH groups in the matrix^35^. For this reason, it can be assumed that molecular chain splitting occurs in the PA5.10 specimens due to hydrolysis. Furthermore, oxidative aging leads to organic compounds like aldehydes (C=O) appear more frequently, which tend to have a low transmittance in the range of 1633 cm^−1^^34,36^. This suggests further degradation of the polyamide’s backbone, due to the molecular chain splitting, most pronounced in the sodium hydroxide solution and 2-propanol stored specimens^34^. Additionally, an emerging peak at 1750 cm^−1^ can be seen subsequent to the storage in sodium hydroxide solution. Lowered transmittance indicates an increase in aldehydes due to oxidation processes^34^.

Figure 5c shows the transmittance spectrum from 1200 to 800 cm^−1^, in which no significant differences from the as-molded specimen exhibit for the specimens stored in distilled water, salt water, soap water, rubbing alcohol, engine oil, acid rain, ethanol and 2-propanol, indicating minimal or no chemical impact on the polyamide’s composition in these bands. The only significant difference can be seen with the sample stored in the sodium hydroxide solution. At 850 cm^−1^, even a new peak appears, which is related to the development of new chemical structures, possibly due to the formation of sodium salts or other degradation products, resulting from the interaction of polyamide with the hydroxide ions^10,14^.

**Fig. 6:**
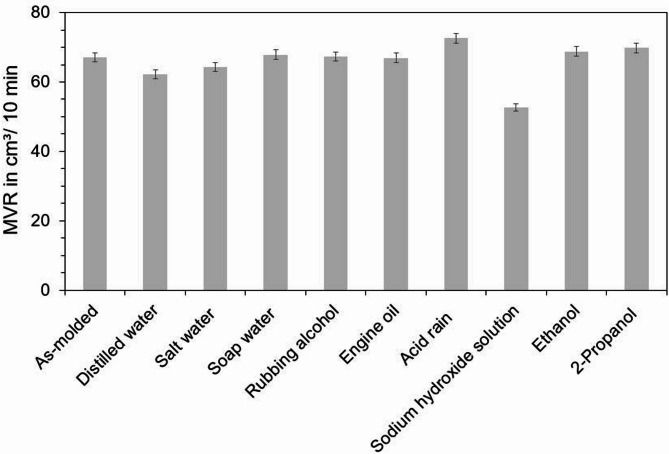
Melt volume rate of the neat PA5.10 specimens subsequent to 168 h of chemical/fluid storage and conditioning. The most noticeable difference can be seen after storage in sodium hydroxide solution, which leads to an increase in viscosity due to the formation of sodium salts.

The determination of the melt volume rate provides insight into viscosity changes of PA5.10 either due to an increased moisture content, changes in crystallinity and hydrogen bonding or aging-related molecular chain splitting processes. This allows the verification of the observations of hydrolytic or oxidative degradation of the FTIR spectrum in Fig. 5. The MVR results of the neat PA5.10 specimens subsequent to 168 h of chemical/fluid storage can be seen in Fig. 6. Compared to the as-molded reference specimens, a decrease in MVR appears in specimens stored in both distilled and salt water, which cannot be observed in soap water. Due to its higher diffusion activity and a lack of dissolved ions, distilled water enables faster and deeper diffusion into polyamide compared to salt or soap water, which both reduce diffusion rates through ionic interactions and altered osmotic gradients. While water uptake typically increases chain mobility due to plasticization, potentially leading to higher MVR, the observed reduction suggests an increased crystallinity and stronger hydrogen bonding, which in turn restricts the melt flow. The most pronounced decline in MVR occurs in specimens stored in sodium hydroxide solution. This finding aligns closely with FTIR spectroscopy results, which indicate significant changes in the polymer’s chemical structure, including clear signs of hydrolytic and oxidative degradation and a strong increase in crystallinity. The alkaline environment likely catalyzes molecular chain splitting processes, particularly at the amide bonds, resulting in structural breakdown which would typically lead to an increase in melt viscosity. Nonetheless, the formation of sodium salts increases the melt viscosity, leading to a decreased MVR. The acid rain stored specimens exhibit the greatest increase in MVR. Due to the high moisture absorption, which can be seen in Fig. 3, as well as structural changes, observed in FTIR measurements in Fig. 5, the increase can be attributed to molecular chain splitting processes and therefore degradation by hydrolysis. In contrast, specimens stored in rubbing alcohol, engine oil, ethanol, and 2-propanol show no significant changes in MVR.

### Mechanical properties

**Fig. 7:**
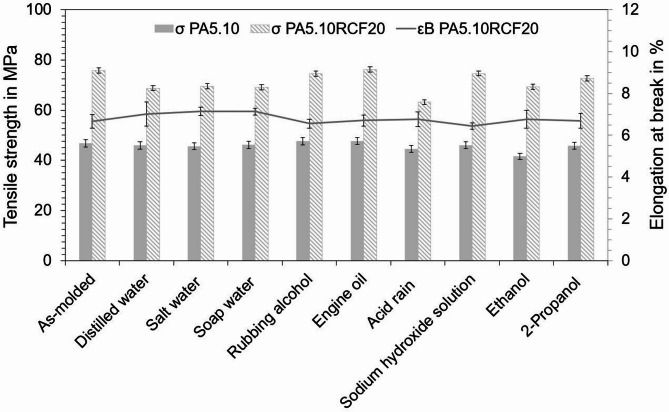
Tensile strength (column charts) and elongation at break (line) of the type 1A test specimens subsequent to 168 h of chemical/fluid storage and conditioning. No significant influence on the elongation at break can be seen across all storage conditions of the RCF-reinforced PA5.10. The tensile strength of the neat PA5.10 is most strongly reduced by storage in acid rain due to hydrolytic degradation processes.

Tensile tests were conducted on the PA5.10 and PA5.10RCF20 type 1 A test specimens in order to assess the mechanical properties. In Fig. 7, the tensile strength is shown as a function of the chemicals/fluids used. Consistent with the current literature, there is an increase in strength with RCF-reinforcement. For both neat and RCF-reinforced PA5.10, after storage in engine oil, rubbing alcohol as well as sodium hydroxide solution, no significant differences are observed when compared to the as-molded type 1 A test specimens. Considering the neat PA5.10 at first, a significant decrease in tensile strength occurs after storage in ethanol. This can be explained by the increased moisture absorption and swelling, visible in Fig. 3, which leads to plasticization and thus to a reduction in mechanical properties^37^. However, for the RCF-reinforced PA5.10, the most significant decrease in tensile strength can be seen after exposure to the acid rain. This observation aligns well with the FTIR results presented in Fig. 5b, where a pronounced hydrolytic and oxidative effect was displayed, suggesting that sulfuric acid as well as the increased moisture absorption has a strong impact on the polyamide’s chemical structure^13^. Since the neat PA5.10 specimens are not notably affected by exposure to acid rain, the decrease in tensile strength might be caused by the degradation of the RCF. In addition, a decrease in tensile strength was also observed in the PA5.10RCF20 specimens stored in the water-based fluids. This is because of the moisture absorption of the PA5.10 and the resulting swelling processes lead to a loss in fiber-matrix-adhesion due to the formation of gaps between the RCF and the matrix, which has been proven in previous studies^9,32,38^. The young’s modulus of the stored specimens behaves in the same way as the tensile strength results, which is why it is not shown separately.

The plotted line in Fig. 7 displays the elongation at break of the RCF-reinforced PA5.10 stored in the chemicals/fluids. As the elongation at break for the neat PA5.10 exceeds 80% even after exposure to various chemicals and fluids, only the RCF-reinforced type 1 A test specimens are shown. However, no significant influence on the elongation at break can be seen across all storage conditions of the RCF-reinforced PA5.10.

Compared to a GFC (PA6GF30), which typically reaches a tensile strength of 160–180 MPa and an elongation at break of 3–4%, the PA5.10RCF20 shows a notably lower tensile strength of 75.79 MPa but at a significantly higher elongation at break of 6.67%^39^. This indicates that while the PA5.10RCF20 does not match the stiffness and strength of PA6GF30, while it offers enhanced ductility, which can be advantageous in applications requiring more flexibility and energy absorption, e.g. in shock-absorbing components in the engine compartment like battery housings or the frontend. Despite the inherently higher sensitivity of the RCF, the bio-based composite the results of the tensile strength exhibit promising chemical resistance, making it suitable for various technical environments in automotive industry.

## Conclusions

This study dealt with the characterization of regenerated cellulose fiber-reinforced bio-based polyamide 5.10 composites subsequent to a storage in various chemicals and fluids used in automotive industry. The results demonstrate that hydrolytic degradation is a key factor affecting the polyamide matrix during the storage. Beyond that, the RCF degrade, especially in acid and alkaline environment, as the glycosidic bonds split because of the presence of functional hydroxyl groups, leading to a reduced fiber strength resulting in fiber ruptures during tensile test. Specimens stored in acid rain, also showed an accelerated water diffusion. Moreover, this caused a significant loss in tensile strength of the neat as well as the RCF-reinforced PA5.10 specimens, as it can be seen in Fig. 7. This is presumably because of the sulfuric acid in the acid rain accelerating hydrolytic chain splitting processes. Furthermore, storage in sodium hydroxide solution revealed distinct molecular changes, including molecular chain splitting processes as it was detected in FTIR analysis in Fig. 5. However, these findings do not align with tensile test results, where no significant reduction occurred subsequent to the sodium hydroxide exposure. This could be due to the fact that the FTIR detected superficial deposits of the sodium hydroxide solution and sodium salts, which also led to an increase in viscosity, seen in Fig. 6. Storage in engine oil showed no significant influence on the PA5.10 or the RCF-reinforced composite. There was an inhibited moisture absorption due to the high viscosity of the oil and no significant effect on tensile strength or elongation at break visible. In the SEM images in Fig. 4d, however, it was observed that the engine oil absorbed into the sample and migrated on the fracture surface subsequent to the tensile test. Storage in the organic solvents ethanol and 2-propanol showed two opposing circumstances. The results of the moisture absorption in Fig. 3 proved that the RCF do not absorb any moisture during the storage in the organic solvents. In addition, however, it has been shown that PA5.10 shows a very strong increase in moisture, especially in ethanol. This can be explained by the fact that, after ethanol evaporation, and due to the subsequent conditioning at standard climate, the hydrogen bonding sites, previously bound with the ethanol, are accessible for water absorption, resulting in a strong increase in moisture.

Compared to conventional GFC, the RCF-reinforced bio-based PA5.10 composites show a differentiated profile of advantages and limitations. While PA6 tends to absorb significantly more moisture than PA5.10, which leads to similar or even stronger hydrolytic degradation effects, the GF are chemically more stable than RCF. As a result, GFC do not exhibit fiber-related degradation such as chain splitting processes in acid environments. Moreover, GF are unaffected by alkaline exposure, whereas RCFs undergo structural weakening, as shown in this study. Although GFC are well-established in industrial applications and offer consistent and reliable mechanical properties, it is associated with a higher density and substantially greater environmental impact. The RCF-reinforced PA5.10, however, offer significant advantages due to their lightweight properties and reduced carbon emissions. However, their susceptibility to aging-induced degradation under certain chemical conditions limits their long-term durability in demanding environments. To counteract these limitations, hydrolysis stabilizers like poly-carbodiimides could be used in the future to reduce the degradation caused by moisture absorption and accelerated degradation in acid or alkaline environment.

## Data Availability

The datasets generated and analyzed in the present study are available in the data repository of the University of Kassel (10.48662/daks-83) on reasonable request.
